# Tin Dioxide Thin Film with UV-enhanced Acetone Detection in Microwave Frequency Range

**DOI:** 10.3390/mi10090574

**Published:** 2019-08-30

**Authors:** Artur Rydosz, Kamil Staszek, Andrzej Brudnik, Slawomir Gruszczynski

**Affiliations:** Department of Electronics, AGH University of Science and Technology, 30059 Krakow, Poland

**Keywords:** gas sensors, acetone detection, microwave application, UV illumination

## Abstract

In this paper, the UV illumination effect for microwave gas sensors based on the tin dioxide was verified. A UV LED with emission wavelength close to the absorption edge of the SnO_2_ gas-sensing layer was selected as the UV source. The developed gas sensors were tested under exposure to acetone in the 0–200 ppm range at room temperature. The sensor’s complex reflection coefficient corresponding to target gas concentration was measured with the use of a five-port reflectometer system exhibiting enhanced uncertainty distribution, which allows for the detection of low gas concentration. The UV illumination significantly emphasizes the sensors’ response in terms of both magnitude and phase for low gas concentrations, in contrast to previously reported results, in which only the reflection coefficient’s phase was affected. The highest responses were obtained for modulated UV illumination.

## 1. Introduction

Acetone (C_2_H_5_OH) is a colorless, mobile, flammable liquid that serves as an important solvent in chemistry and industry. Recently, it has become attractive for biomedical applications, where it is considered a biomarker of diabetes, due to its presence in exhaled breath in various concentrations for healthy and diabetic patients [1,2,3,4,5]. Patients with diabetes tend to have higher acetone levels (1.25–2.5 ppm) in their breath than healthy people (0.2–0.8 ppm). On the other hand, the inhalation of acetone at higher concentrations (200–2000 ppm) may lead to hepatotoxic effects, causing liver damage [6]. Therefore, it is essential to detect acetone at lower and higher concentrations. The detection at higher concentrations is covered by commercially available sensors, e.g., TGS Figaro [7], but detectors able to detect acetone in the sub-ppm range are still under investigation. In the last few years, a number of papers have focused on enhanced acetone detection, utilizing various methods, such as optical detection [8,9,10], electrochemical sensors [11,12,13], metal oxides (MOXs)-based sensors [14,15,16,17,18,19,20], and analytical systems [21,22,23,24,25]. Microwave-based gas sensors with various gas-sensitive layers, including organic layers [26,27] and various MOXs layers [28,29,30], were investigated by the authors as well. The recently obtained results have shown that microwave gas sensors based on metal oxides can easily be utilized for acetone detection in the ppm range at room temperature [28,29,30]. However, for such sensors, the response/recovery time(s) at room temperature is longer than for conventional applications, where the operating temperature is usually in the 300–500 °C range. To overcome this limitation and to increase the gas sensor response, a UV illumination can be utilized. 

Recently, Khan et al. [31] presented the development of a toluene detector based on deep UV absorption spectrophotometry. The setup was tested for different toluene concentrations (10–100 ppm) and a linear relationship between gas concentration and the absorbance was observed. The sensitivity and selectivity of the setup can be improved by coupling it with a preconcentration unit, e.g., [32,33,34]. Bastates et al. [35] measured the effect of UV illumination (369 nm, 17 mW) on the detection of ammonium nitrate (NH_4_NO_3_) by a ZnO-coated nanospring sensor operated at room temperature [35]. The investigation results showed that UV illumination reduces surface band bending and reduces the sensor recovery time between detection events by shortening the decay time of the signal [35]. A good review of light-activated metal oxide gas sensors was delivered by Xu and Ho [36]. The authors reviewed the progress of light-activated conductometric gas sensors based on metal oxides, such as pure metal oxides, 1D nanostructures, and porous nanostructures. They confirmed that light intensity at a specific wavelength can change the sensing response and even tune the device’s selectivity, however, the correct intensity level, as well as film thickness, must be investigated for each MOX separately [36]. 

In this paper, a microwave gas sensor based on the tin dioxide (SnO_2_) gas layer with UV illumination is investigated for acetone detection in the 0–200 ppm range at room temperature. The gas-sensing properties of SnO_2_ at microwave frequencies were previously confirmed and presented in [30]. The obtained results strongly confirm that the UV illumination increases the developed sensor’s sensitivity for acetone, allowing for the detection of lower gas concentrations. Moreover, a linear relationship between acetone concentration and magnitude/phase changes of the sensor’s reflection coefficient was observed. 

## 2. Materials and Methods

### 2.1. SnO_2_ Deposition Technology 

Tin dioxide (SnO_2_) thin films were deposited in RF (radio frequency, 13.56 MHz) mode from the Sn metallic target by applying reactive sputtering under a mixture of 80% argon and 20% oxygen and by applying GLAD (glacing angle deposition ). The base vacuum and deposition vacuum were 1 × 10^−5^ mbar and 2 × 10^−2^ mbar, respectively. The deposition temperature was set to 200 °C and deposition time was adjusted to deposit various thicknesses (50 nm, 250 nm, and 500 nm), with a constant power of 50 W. The films’ thicknesses were measured post-process using a TalyStep profilometer (Taylor Hobson, Leicester, UK). The fabricated sensors were tested for gas-sensing applications and the highest responses were obtained for 250 nm thin films [30], therefore these sensors were investigated for the UV illumination effect. The sputtering deposition system was presented previously in [30]. 

### 2.2. Microwave Measurements

As was shown in [30], the above described SnO_2_ layer changes its permittivity in the microwave frequency range when exposed to acetone. This phenomenon has been used for indirect acetone concentration measurement with the use of the microwave measurement system reported in [37], operating at a frequency of 2.4 GHz. This system is composed of a microwave sensor with the SnO_2_ as a gas-sensing layer and a dedicated five-port reflectometer for measuring the sensor’s response.

**Figure 1:**
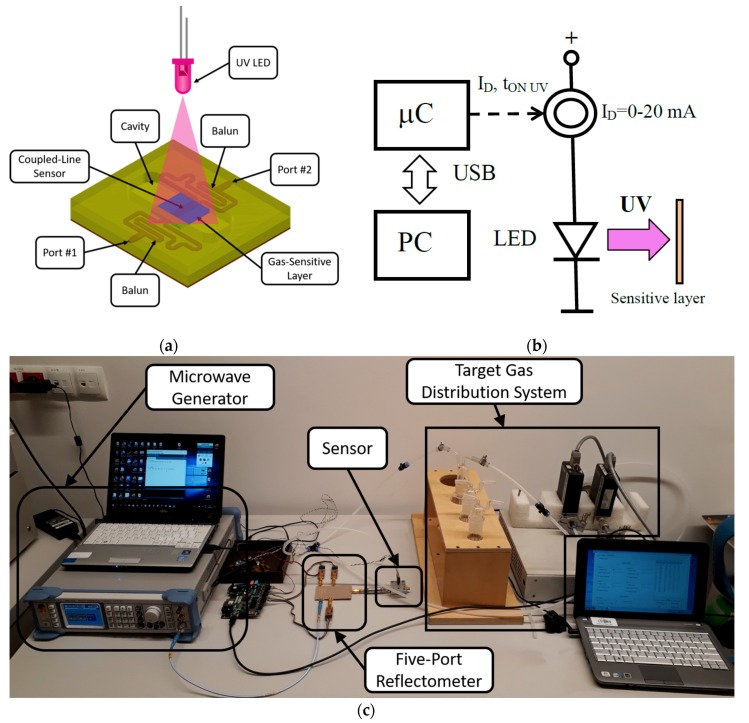
The microwave gas sensor system: (**a**) sketch of the microwave gas sensor; (**b**) sketch of the UV illumination system; (**c**) photo of the gas-sensing system.

The microwave sensor is presented in Figure 1a. It was composed of two baluns, between which a coupled-line section covered with a SnO_2_ layer was inserted. These baluns ensured the odd-mode excitation of the mentioned coupled-line section, enhancing the sensor’s sensitivity. When exposed to acetone present in the cavity, the SnO_2_ layer changed its permittivity, affecting the electromagnetic field distribution along the coupled-line section, which in turn changed the sensor’s transmission coefficient. For further sensitivity increase, the sensor was used in a single-port configuration with port #2 left opened. In such a case the reflection coefficient seen at port #1 (the measured value) is approximately equal to the transmission coefficient’s squared, since a microwave signal propagates through the sensor from port #1 to port #2 and is reflected. As a result, such a sensor configuration doubles the impact of the permittivity change on the measured reflection coefficient. To obtain good measurement quality, the reflection coefficient measurement was realized with the use of the recently developed five-port reflectometer [37]. It exhibited significantly enhanced measurement uncertainty for the reflection coefficient’s range, corresponding to the utilized sensor’s reflection coefficient (magnitude equal to 0.8 ± 0.2 and phase equal to 180° ± 15°) [37], with respect to the classic reflectometers optimized for all reflection coefficients (magnitude not exceeding 1, arbitrary phase). It consisted of a five-port passive power distribution network, signal source, and three microwave power detectors, the readings of which were translated to the measured complex reflection coefficient. Since the utilized system was able to measure both the magnitude and phase of the sensor’s reflection coefficient, the gas-sensor response can be defined twofold, i.e., as magnitude difference $\Delta \left|s_{11}\right|$ and phase difference $\Delta arg\left[s_{11}\right]$ of the reflection coefficients measured under exposure to target gas *s*_11 gas_ and air *s*_11 air_:

(1) $$\Delta \left|s_{11}\right|=\left|s_{11\;gas}\right|-\left|s_{11\;air}\right|;$$

(2) $$\Delta arg\left[s_{11}\right]=arg\left[\frac{s_{11\;gas}}{s_{11\;air}}\right].$$

The entire measurement system is shown in Figure 1c.

### 2.3. Gas-sensing Protocol with UV

**Figure 2:**
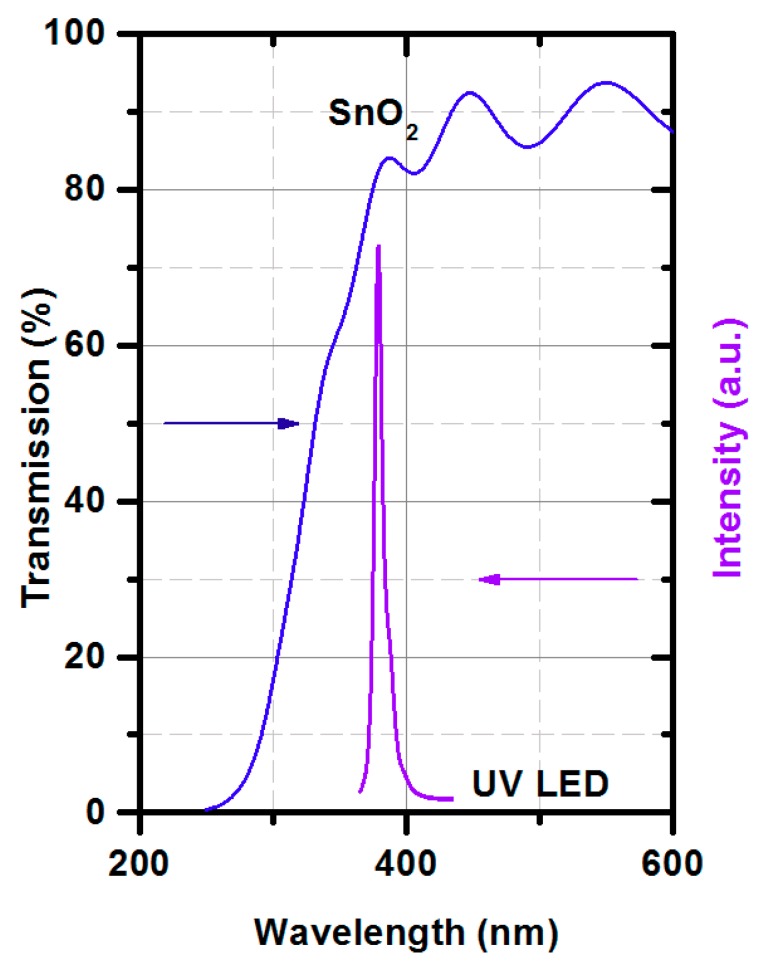
The SnO_2_ transmission with the intensity of the UV LED diode (OSV2YL5111A) in the function of wavelength.

Figure 1b shows the UV LED supply circuit. The UV source was a UV LED (OSV2YL5111A) with λ = 375 nm. Figure 2 shows the transmission of the SnO_2_ thin film working as the gas-sensitive layer and emission of the UV LED. The transmission was measured by a Lambda 19 Spectrophotometer (Perkin-Elmer, UK) and the UV LED emission by a monochromator SPM-2 (Carl-Zeiss, Jena, Germany). The λ (375 nm) was chosen to be in the absorption edge of the gas-sensitive layer, as it was presented in Figure 2. 

To control the diode current ID (0–20 mA), a microcontroller Atmega 328 was used with a dedicated software on PC via a USB port. The measurements were conducted in two modes: CW (continuous wave) and PWM (pulse width modulation). The gas-dosing system was based on the mass flow controllers 1179B (MKS Instruments, New York, NY, USA), Dreschel bottles, and gas canisters (Air Liquid, Krakow, Poland). The data was collected by dedicated software and further processed by developed algorithms. All experiments were conducted at room temperature and 50% relative humidity level, controlled by an AC system in the laboratory. 

## 3. Results and Discussion

### 3.1. Gas-sensing Characteristics for Continouse UV Radiation

**Figure 3:**
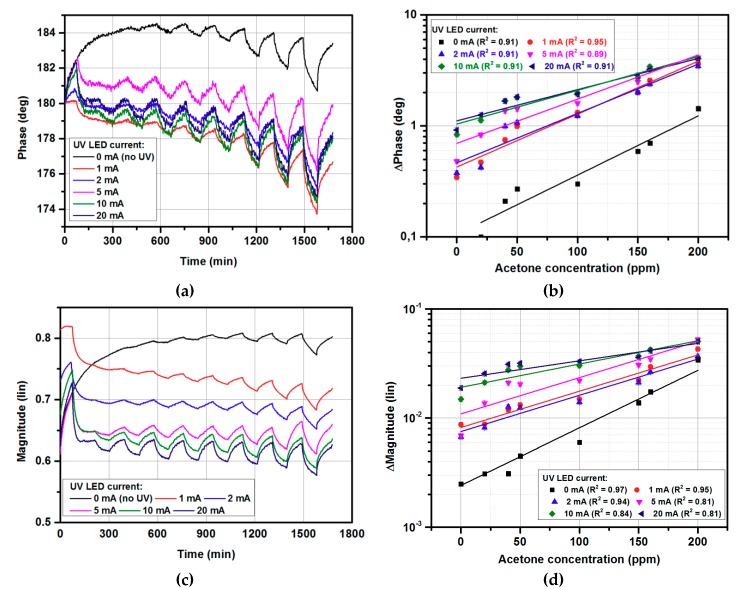
The gas-sensing characteristics under exposure to acetone in the 0–200 ppm range: (**a**) phase changes in time domain; (**b**) calibration curve of phase changes; (**c**) magnitude changes in time domain; (**d**) calibration curve of magnitude changes.

Figure 3 shows the gas-sensing characteristics under exposure to 0–200 ppm acetone at continuous UV illumination with various LED diode currents. The developed system measured both the phase and magnitude of the sensor’s reflection coefficient. Figure 3a,c show the phase and magnitude changes under exposure to 0–200 ppm of acetone, respectively. The lowest acetone concentration used during the measurements was 20 ppm, based on the limitation of the gas distribution system, for which the response was 0.02 of the magnitude change and 1° of phase change (at 10 UV CW illumination). On the other hand, the magnitude and phase noise level were equal to 0.0033 and 0.21°, respectively (calculated as 3σ values). As can be observed, the obtained values were significantly larger than the noise level for both magnitude and phase, hence a lower concentration of acetone than 20 ppm could be measured. Nevertheless, for such low concentrations, the long-term drift seen in both measured magnitude and phase responses becomes dominant and needs to be cancelled to obtain more accurate results. The measurement uncertainty was below 5%, and error bars are represented by size of the measurement points.

As can be observed, the UV illumination significantly emphasized the sensor response in terms of both magnitude and phase for low gas concentrations, in contrast to previously reported results, in which only the reflection coefficient’s phase was affected [37]. However, this effect decreased for higher concentrations, for which an emphasis was not required. Hence, UV illumination increased the sensor’s sensitivity and simultaneously held the measured reflection coefficient in the area described in Section 2.2, for which the utilized five-port reflectometer was optimized. Thanks to this effect, low measurement uncertainty was preserved for all the measured gas concentrations. As seen in Figure 3b,d, the magnitude changes were of higher quality than the phase changes and could be easily used as gas sensor response signal, defined by (2) in Section 2.2. Increasing the diode current increased the gas sensor’s response, however, the UV LED current higher than 10 mA did not provide further sensitivity enhancement, therefore 10 mA was set for further experiments, to restrain the power consumption. Furthermore, Figure 2b shows that applying UV illumination even with a 1 mA diode current significantly improved the gas sensor’s response in comparison with no UV illumination. 

### 3.2. Gas-sensing Characteristics for Modulated UV Illumination

**Figure 4:**
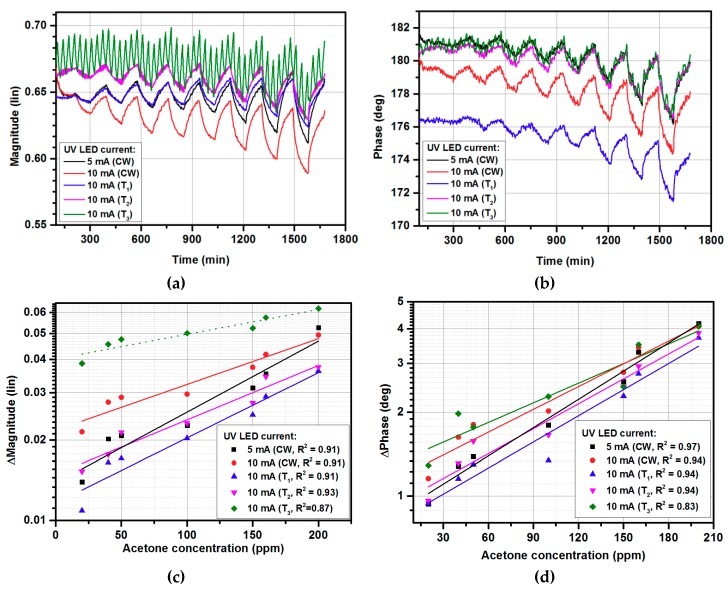
The gas-sensing characteristics under exposure to acetone in the 0–200 ppm range: (**a**) magnitude changes in time; (**b**) phase changes in time; (**c**) calibration curve for magnitude changes; (**d**) calibration curve for phase changes.

After measurement with continuous wave (CW) UV illumination at various UV currents (Section 3.1), the developed sensor was tested under exposure to acetone and three various periods of UV switching were applied. The periods were T_1_, T_2_, and T_3_, where the UV light was switched ON/OFF (current amplitude was 10 mA) for 20 s, 200 s, and 2000 s, respectively. Figure 4 presents the obtained results. As can be observed, the magnitude and phase changes for UV modulation with T_1_ and T_2_ periods had lower values in comparison with 5 mA continuous wave illumination. The modulation with the 2000 s period (T_3_) affected the magnitude measurements, where the switching effect is visible. The switching effect with T_1_/T_2_ periods was unnoticeable. On the other hand, T_3_ was ~1/3 of the exposure time (5400 s), hence, for a single acetone concentration, the UV light was switched ON/OFF around three times (Figure 4a). Therefore, to calculate the sensor’s sensitivity using (1), the min/max values must be taken considering three ripples, in contrast to a single ripple occurring for every other case. A reduction in UV illumination cycles number responded with higher gas sensor responses (Figure 4c, dotted line), calculated from the magnitude signal’s envelope. Moreover, the limit of detection for sensors with UV signal modulation close to the exposure time was higher than for continuous wave modulation.

### 3.3. Gas-sensing Characteristics for Pulse Width Modulated (PWM) UV Illumination

**Figure 5:**
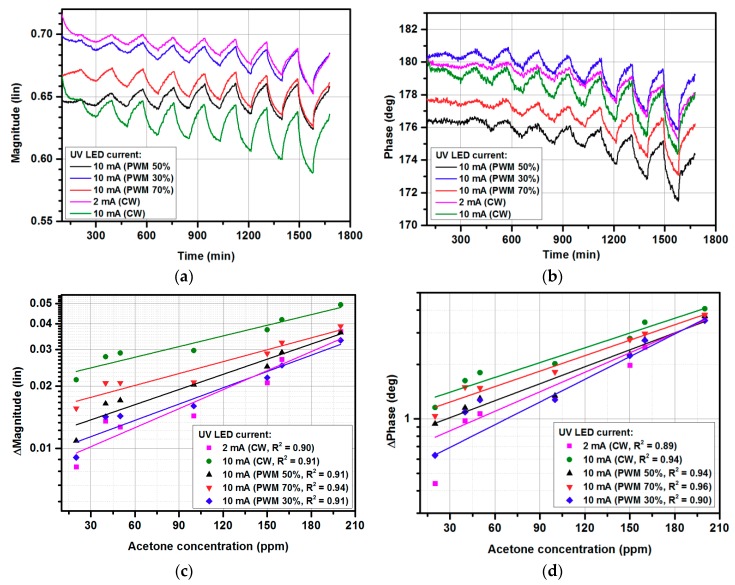
The gas-sensing characteristics under exposure to acetone in the 0–200 ppm range: (**a**) magnitude changes in time; (**b**) phase changes in time; (**c**) calibration curve for magnitude changes; (**d**) calibration curve for phase changes.

The developed sensors were tested under exposure to acetone and pulse width modulation (PWM) with various duty cycles: 30%, 50%, and 70%, and 10 mA amplitude. As can be observed in Figure 5, the duty cycles correspond to continuous waves with 3 mA, 5 mA, and 7 mA, respectively. It has to be underlined that the period of duty cycles was equal to T_1_ from Section 2.1. The magnitude/phase measurements exhibited slight drift, which is most likely due to local temperature and humidity changes and synthetic gas impurities. The total time for one series of measurements was 28 h.

**Figure 6:**
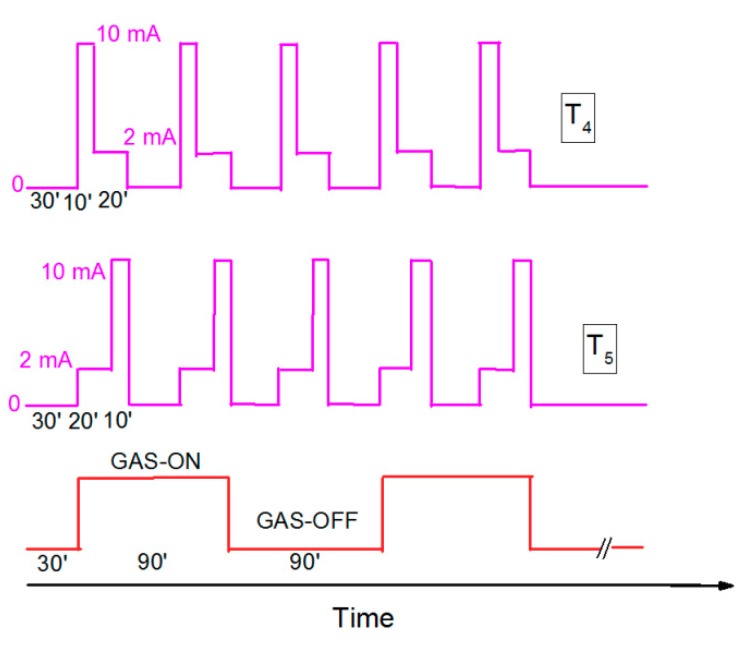
The PWM sequences named T_4_ and T_5_, and gas-sampling sequence.

**Figure 7:**
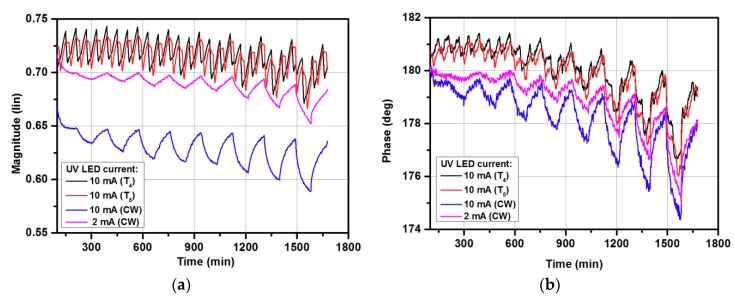
The gas-sensing characteristics under exposure to acetone in the 0–200 ppm range: (**a**) magnitude changes with various PWM cycles (T_4_, T_5_); (**b**) phase changes with various PWM cycles (T_4_, T_5_).

Figure 6 shows two UV illumination sequences (T_4_, T_5_) with a gas-sampling sequence. The gas-sampling process started with 30 min without target gas (only synthetic air), then 90 min periods with ON/OFF gas were set. The various gas concentrations were used as presented in Figure 4. The UV illumination switching was preset to start with ‘high’ UV signal for 10 min. and then ‘low’ for 20 min, and 30 min without UV light (sequence T_4_), or to start with ‘low’, ‘high’, and without (sequence T_5_). The idea behind using various PWM sequences instead of CW signals was to reduce power consumption, and to introduce the UV at the beginning and at the end of exposure to gas and in the middle of the no-gas period. Such a method was proposed to verify the influence of UV illumination for gas-sensing characteristics. In sequence T_5_, the UV light was applied with high signal (10 mA) at the same time as when the sensor was exposed to the target gas, which should increase the sensitivity, and the low signal (2 mA) was kept, to stabilize the response at lower power consumption. The same method was repeated at the end of the gas-sampling procedure. After dosing the target gas, the synthetic air was introduced to the gas chamber without UV illumination, which should react with a longer recovery time. To verify this hypothesis, the UV illumination was again switched on in the middle of exposure to synthetic air. The sequence was repeated for all target gas concentrations. Sequence T_5_ was prepared in reverse to T_4_, where the low signal was first applied and then a high signal. As can be observed, the magnitude (Figure 7a) and phase (Figure 7b) were higher in comparison with the 2 mA and 10 mA continuous wave, however, the sensor response defined as magnitude/phase changes did not change significantly. Further investigations are needed to find the optimal working conditions, where a continuous UV signal can be successfully replaced for modulated ones and where not only the UV LED current amplitude will be modulated but also the wavelength of the UV source.

## 4. Conclusions

Microwave gas sensors operated at microwave frequencies, such as 2.4 GHz and at room temperature, can be applied in many various applications. Previously, various metal oxides have been proposed as a gas-sensitive layer for acetone detection [30]. The conducted research has confirmed that the sensitivity of the microwave gas sensors based on SnO_2_ can be further improved by applying UV illumination, which emphasizes the sensor’s response to lower gas concentrations. Simultaneously, it does not magnify this response for higher concentrations, preserving the measured reflection coefficient in the range for which the measurement uncertainty of the utilized system is optimized. As a result, a good measurement quality has been obtained for a wide range of gas concentrations. Various experimental conditions were tested. The highest sensitivity was obtained for a UV (375 nm) current of 10 mA at continuous wave. However, further investigation is needed to find the optimal conditions with PWM modulation and to select the UV diode wavelength in terms of various metal oxide layers.
